# Should I Buy Groceries or Get a Diagnostic Scan?

**DOI:** 10.1089/heq.2024.0111

**Published:** 2025-03-19

**Authors:** Brad Isaacson, Emma Greally

**Affiliations:** ^1^ Tellica Imaging, LLC, An Intermountain Health Company, Salt Lake City, Utah, United States.; ^2^ Harvard College, Cambridge, Massachusetts, United States.

**Keywords:** imaging, MRI, CT, cost, IDTF

## Abstract

The economic burden of medical care is shared (often disproportionately) between patients, health care systems, and payers. Diagnostic scans in particular provide valuable information for providers; however, imaging is often performed in high-cost settings. Independent Diagnostic Testing Facilities have emerged as viable options for patients, but care is often fragmented and may lack sufficient quality standards. To change health care, bold steps are needed to maximize value-based care, enhance access, and ensure affordability for communities. If this fails to occur, for many more years, we will unfortunately hear “should I buy groceries or get a diagnostic scan?”.

## Narrative

The United States (US) is at a critical tipping point with medical expenditures totaling over $4.5 trillion, or 17.3% of gross domestic product (GDP).^1^ With the average American expected to spend approximately $13,500 annually for their care,^2^ patients are forced to ask themselves, “should I buy groceries or get a diagnostic scan?” This heartbreaking question epitomizes the economic hardships and rising costs of care that exist within the US health care system.

Advanced imaging such as computed tomography (CT), magnetic resonance imaging (MRI), and positron emission tomography serves an invaluable role in diagnostic and treatment care. However, a scan can exceed $10,000 for a simple 30-minute appointment. The financial burden of radiology has increased significantly in the 21st century as life expectancy in the US continues to rise, patients demand a high quality of life, and consumers rely more on high-deductible health plans to defray expensive premiums.^3^ Recent literature that reviewed out-of-pocket (OOP) expenditures in imaging demonstrated that between 2000 and 2019, there was an 89.9% increase for consumers, with the trend increasing exponentially following the COVID-19 pandemic.^4^ The epidemiological study concluded that “among various essential in-network benefits across health insurance plans nationwide (e.g., laboratory testing and specialist care), patient OOP costs for advanced imaging recently ranked number one.”^4^

Although the passage of the Patient Protection and Affordable Care Act has been a positive first step to defray costs, particularly with radiology, the barrier to receiving timely imaging services remains due to the lack of transparency in health care, uncertainty of covered services, and unclear in-network options. In fact, many health care plans include costly coinsurance/copayments for out-of-network services, which can result in 100% financial responsibility for patients. With one third of Americans reporting financial medical distress, advanced imaging remains one of the most frequently ordered exams with no sign of relenting. For example, there were 3 million CTs ordered in 1980, but over 80 million presently in the US.^5^

Policies such as the “No Surprises Act” have emerged at the federal level to assist those seeking emergent and noncritical care from out-of-network providers at in-network facilities (including ancillary services such as radiology, pharmacy, laboratory, and air ambulance). However, 45% of Americans still fear major health events will leave them bankrupt, and 77% are concerned that health care costs will cause lasting damage to the US economy.^6^ Furthermore, the uninsured are less likely to seek care altogether, and when they do, they often utilize expensive treatment sites, such as emergency departments, with the average visit costing $2500 out of pocket.^7^

While imaging for most is considered episodic (e.g., a shoulder impingement from the gym, abdominal pain from feeling unwell, a fractured ankle from a fall, etc.), chronic conditions such as multiple sclerosis (MS) require MRIs every 6 months to monitor disease progression. Studies have shown that this cohort has a higher percentage of financial hardship than peers without this disease, and their debt accumulates over time. With an annual estimated total cost of care for an individual with MS of $88,000^8^ and 2.8 million people suffering worldwide (1 million of which are in the US),^9^ medical imaging is both a necessary tool and an unjust financial obligation for syndrome management.

To reduce costs for patients, health care systems, and insurance payers, outpatient imaging has emerged as an option in many communities across the US. The economics are simple: by moving advanced imaging to a more affordable site of care with lower indirect costs, these savings can be returned to consumers through reduced imaging fees. However, this is not always the case, as many imaging chains are still private equity financed where investors expect nearly 25% of profits. When the authors of this article called several outpatient imaging businesses in a large city to compare prices, quotes ranged between $1500 and $2600 for a basic brain MRI with and without contrast, a common scan needed to assess MS. With the national median household income in the US around $75,000, the recommendation of two scans per year at these locations would equal about 7% of yearly income for a patient. Most concerning is that many outpatient sites labeled Independent Diagnostic and Testing Facilities do not match the quality of hospital-based care in order to offer “discount” imaging (e.g., using a singular general radiologist performing all professional interpretations compared to numerous specialized fellowship-trained providers, having machines that are older/outdated, not offering standardized protocols, limiting contrast cases). Sacrificing safety and quality at the expense of imaging prices and affordability should never have to occur for patients seeking care.

Michael Porter famously shared in 2006 that redefining health care could be viewed as a function of outcomes divided by the cost per patient to achieve a desired result. Since then, “value-based care (VBC)” has continued to permeate mainstream discussions on health care innovation, but many still struggle to understand its true meaning: some institutions have redefined value as a product of quality and service divided by the cost, but replicating hospital-based services at an affordable price in other venues has remained limited. To engage in VBC effectively and efficiently, the medical/health care community must seek to take a consumer-driven mindset while emphasizing access, affordability, quality, and care. Medical imaging lends itself to a shift toward VBC given that providers can perform diagnosis through teleservices as well as more affordable venues external to the health care system, such as outpatient imaging locations that reduce costs for patients while maintaining a high standard of care, both of which are modalities that are Food and Drug Administration regulated. With national health care expenditure expected to grow 5.4% between 2019 and 2028, representing 19.7% of US GDP,^10^ our nation’s health care must find a way to disrupt our costly, inaccessible, and frictional care delivery system to eliminate the question, “should I buy groceries or get a diagnostic scan?”

## Abbreviations Used

CT: Computed Tomography
GDP: Gross Domestic Product
MRI: Magnetic Resonance Imaging
MS: Multiple Sclerosis
OOP: Out of Pocket
US: United States
VBC: Value-Based Care
